# Comparative insights into soybean and other oilseed crops’ defense mechanisms against *Sclerotinia sclerotiorum*

**DOI:** 10.3389/fpls.2025.1616824

**Published:** 2025-06-24

**Authors:** Nick Talmo, Ashish Ranjan

**Affiliations:** Department of Plant Pathology, University of Minnesota – Twin Cities, St. Paul, MN, United States

**Keywords:** *Sclerotinia sclerotiorum*, resistance mechanisms, effectors, organic acids, host pathogen interaction, soybean, canola, sunflower

## Abstract

*Sclerotinia sclerotiorum* is a devastating fungal pathogen capable of causing substantial yield loss on a wide range of agronomically important crops worldwide. *S. sclerotiorum’s* impressive virulence across its broad host range is primarily due to the abundance of pathogenic strategies at its disposal. These pathogenic strategies include the use of organic acids, hydrolytic enzymes, and various effector molecules that work in concert during host attack. While plants have evolved sophisticated defense mechanisms, complete resistance to *S. sclerotiorum* remains elusive among the more than 400 known plant hosts. Among these hosts, soybean, canola, and sunflower are the most important oilseed crops severely affected by *S. sclerotiorum* infection, which can result in 94% crop loss in extreme cases. Current management strategies rely on chemical fungicides, crop rotations, and partially resistant varieties, albeit with varying levels of success. Despite extensive research on individual host-pathogen interactions, there is a notable gap in comparative studies exploring defense mechanisms across plant families. This review seeks to address this gap by providing an overview of known defense strategies against Sclerotinia stem rot (SSR) in soybean and canola, as well as head rot (SHR), mid-stalk rot (MSR), and basal stalk rot (BSR) in sunflower. By identifying commonalities and differences among distantly related hosts, this comparative analysis aims to deepen our understanding of key plant defense strategies against *S. sclerotiorum*, thereby highlighting areas requiring future research.

## 1 Introduction

**Figure 1:**
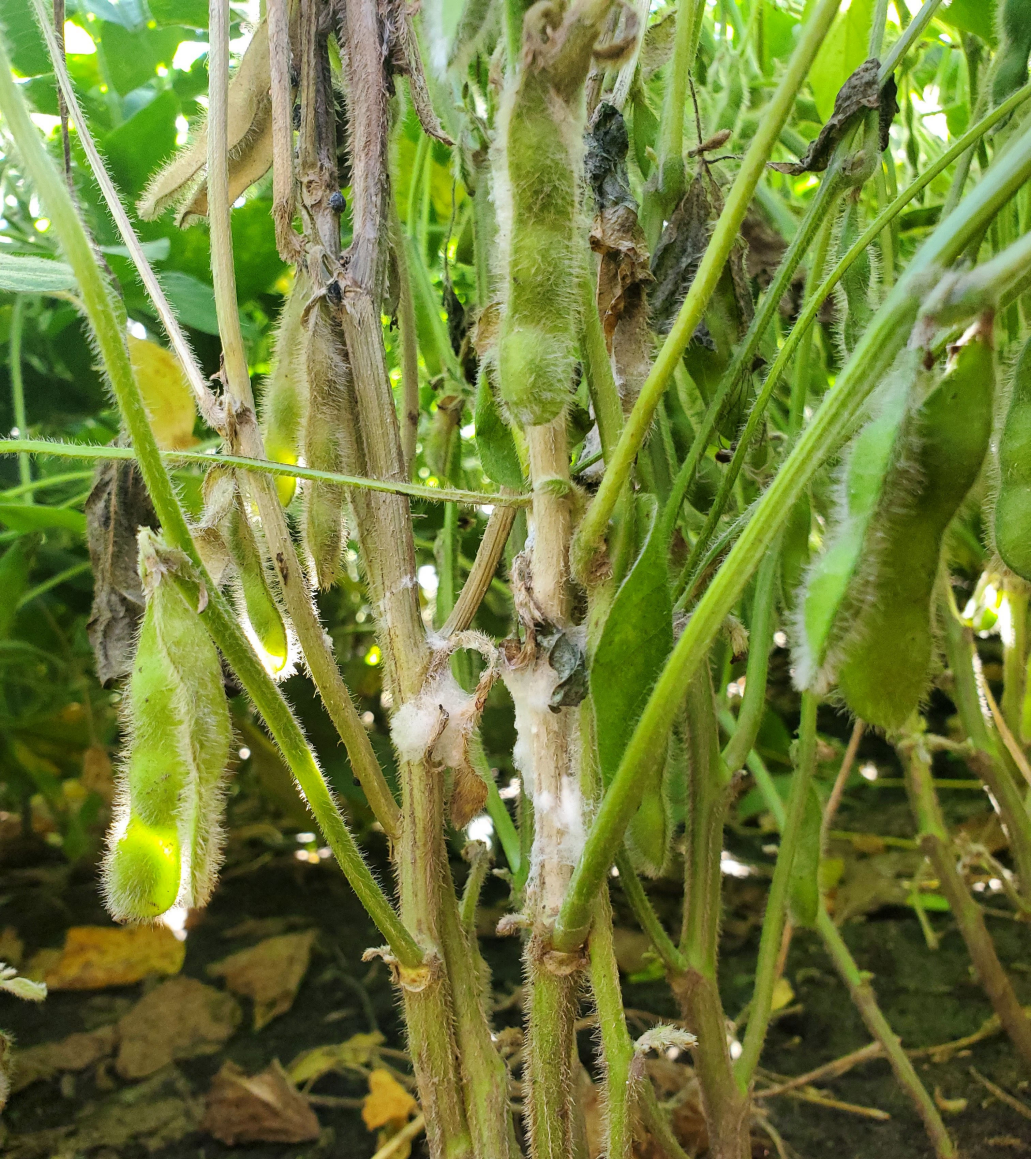
White mold disease appearance on soybean infected with *Sclerotinia sclerotiorum*.

*Sclerotinia sclerotiorum* is a hemibiotrophic, filamentous ascomycete that is a formidable plant pathogen worldwide. It is currently estimated to infect 425 plant species comprising 74 families, mainly within dicotyledonous plants, although a few monocotyledonous species are also affected (Derbyshire et al., 2022). *S. sclerotiorum* was first described by Libert in 1837 (Libert, 1837). In 1945, Whetzel defined the pathogen as the primary species of the genus *Sclerotinia* (Whetzel, 1945). This soil-borne fungal pathogen is named for its durable, melanized resting structures known as sclerotia, which germinate myceliogenically or carpogenically to form hyphae or fruiting bodies, respectively. Hyphae are hyaline, septate, and multinucleate and develop in a branched pattern (Maxwell et al., 1972; Willetts and Wong, 1980). The fruiting bodies of *S. sclerotiorum* are known as apothecia, which are cup-shaped and sit atop a stalk that rises from the germinated sclerotia (The Canola Council of Canada, 2020). The diseases caused by *S. sclerotiorum* are often referred to as white mold due to the production of cottony masses on infected tissues (**Figure 1**). White mold is most devastating in temperate regions throughout the world, although it has been reported on every continent except Antarctica (Seiler et al., 2017). White mold is a formidable pathogen of many crops and ranks globally among oilseed crops as one of the most significant, economically devastating fungal pathogens (Gulya et al., 2016; Roth et al., 2020; Zheng et al., 2020).

Three of its most economically important oilseed crop hosts are soybean, canola, and sunflower. Yield reductions among these hosts vary depending on environmental conditions and management practices. In severe cases, such as the one seen in Wisconsin in 2009, it was estimated that 10% of the soybeans grown that year were lost to white mold (Crop Protection Network, 2023). The National Sclerotinia Initiative reports that combined soybean, canola, and sunflower losses can be as high as $424 million in the United States in the year 2021. Individually, losses have totaled $300 million in soybean, $24 million in canola, and $100 million in sunflower (U.S. Department of Agriculture-Agricultural Research Service, 2022).

*S. sclerotiorum’*s devastating nature is the result of numerous compounding factors. The pathogen’s polyphagous diet and widespread distribution are likely due to the multitude of phytotoxic metabolites, hydrolytic enzymes, and effectors that it uses during attack of its host. *S. sclerotiorum’s* ability to infect such a wide range of hosts is not fully understood. However, current understanding regarding its broad host expansion suggests preadaptation of *Sclerotinia* spp., in which the species developed virulence factors capable of targeting highly conserved plant defense mechanisms (Derbyshire et al., 2022).

Although this pathogen has adopted many complex and robust virulence mechanisms, plant hosts have also evolved an array of defense strategies in this evolutionary arms race. Among these divergent hosts, many conserved and overlapping defense response pathways exist, including the production of polygalacturonase-inhibiting proteins (PGIPs), antimicrobial phytoalexins, and flavonoids. However, variation and divergence among these orthologous systems are widespread, and the evolution of clade or family-specific defenses is not uncommon. For example, glyceollins comprise a class of phytoalexins specific to the genus *Glycine* that have demonstrated inhibition of *S. sclerotiorum* growth in culture (Kim et al., 2010b; Lygin et al., 2010). Glucosinolates, a secondary metabolite produced strictly by crucifers, have also been shown to increase the defense responses of *B. napus* when challenged with SSR (Bednarek et al., 2009).

Conversely, in sunflower, *S. sclerotiorum* does not cause one disease. Instead, it can result in one of three different diseases depending on the location of infection. These diseases include basal stalk rot (BSR), mid-stalk rot (MSR), and head rot (SHR), demonstrating the pathogen’s ability to infect more than just stem tissues in this host. Understanding what makes all plant organs of sunflower susceptible while other hosts are able to prevent root infection requires comprehensive comparative studies of the diverse hosts’ physiological and molecular resistance and susceptibility mechanisms against *S. sclerotiorum*.

This review discusses past and recent progress in oilseed crop management against SSR pertaining to the many virulence mechanisms of *S. sclerotiorum* and the defense mechanisms employed by these three oilseed crop hosts. By exploring individual pathosystems and identifying the similarities and differences among host responses, we hope to motivate comprehensive comparative studies to better understand how to develop resistance against *S. sclerotiorum.*

## 2 Life cycle and pathogenic strategies of *Sclerotinia sclerotiorum*

**Figure 2:**
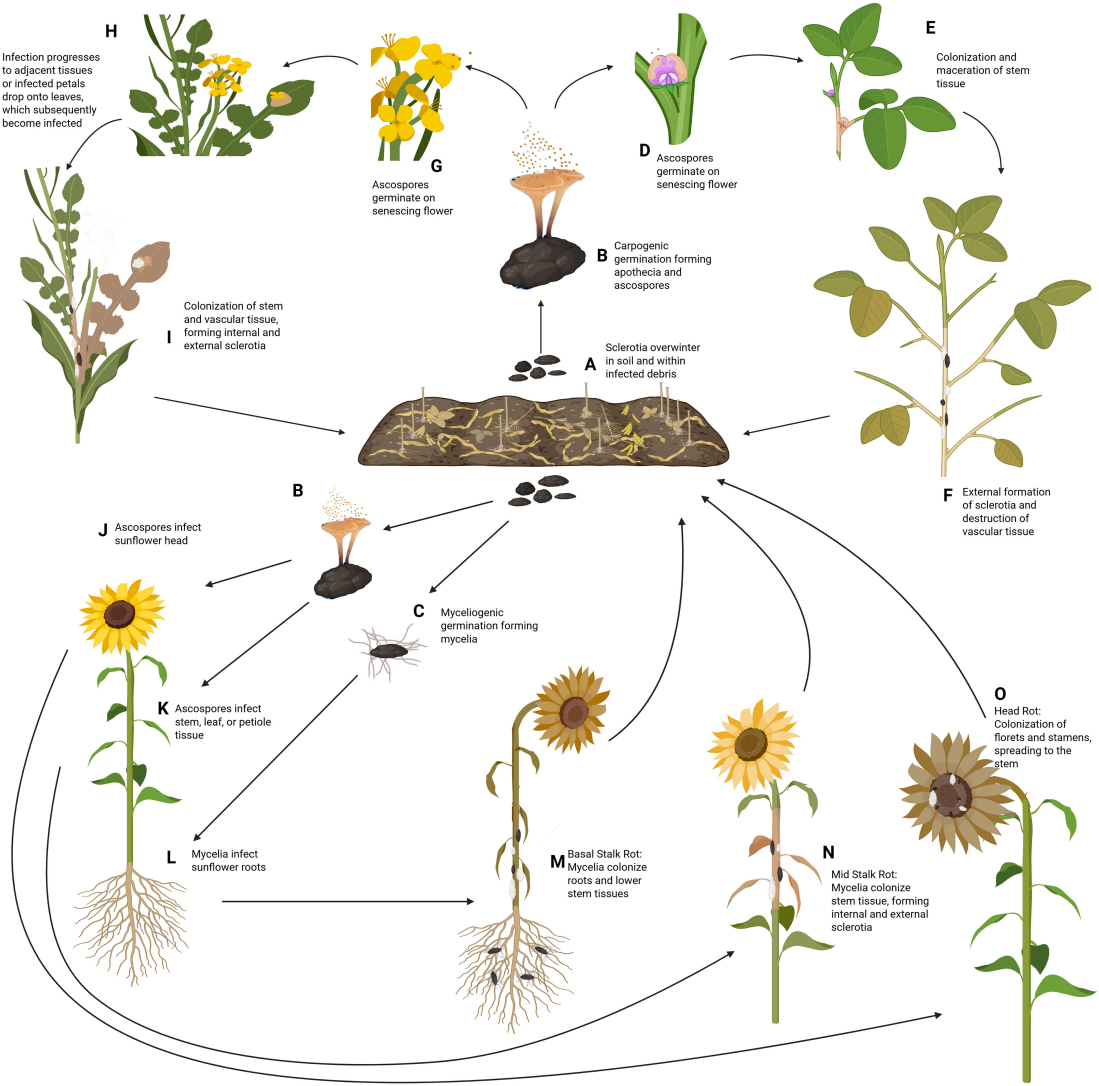
Disease Cycle of *S. sclerotiorum* on soybean, canola, and sunflower. **(A)** *S. sclerotiorum* survives in the soil and within infected debris as sclerotia, remaining viable for up to eight years. **(B)** Under conducive conditions, these sclerotia germinate carpogenically in the spring, to form a fruiting structure known as apothecia. Each apothecia can produce more than 10 million ascospores which are then wind disseminated to the host plant. **(C)** Alternatively, sclerotia can germinate myceliogenically, producing hyphae that will grow outward in the soil towards plant roots. In soybean **(D)**, under natural conditions, the ascospores will land on senescing flowers, which release nutrients utilized by the germinating ascospore. Following germination, germ tubes form, and mycelia begin to colonize the plant tissues. **(E)** Mycelia will continue spreading from the point of infection, colonizing and macerating the stem tissues. **(F)** The water-soaked lesions will begin to dry out as the pathogen radiates from the point of infection, girdling the stem and replacing the pith with sclerotia, which will be deposited back into the soil for subsequent seasons. In canola **(G)**, like soybean, the ascospores are deposited on senescing flowers during the rosette phase, where the released nutrients are utilized for hyphal formation. **(H)** Following infection of the flower, the pathogen can spread to the stem directly from the flower. Alternatively, the flower petals may detach before reaching the stem. However, if the infected petal lands on a leaf below, the mycelia will penetrate the leaf and spread to the remainder of the plant. **(I)** Further colonization of the stem results in a destruction of vascular tissue, death of the plant, and formation of sclerotia internally and externally, which can be deposited into the soil for the following year. In sunflower **(J)**, ascospores can be deposited on the flower head, where they utilize nutrients from saccharose glands to germinate. **(K)** The ascospores can also be deposited and initiate infection on the stems, leaves, and petioles of the plant. **(L)** Alternatively, the sclerotia can directly produce hyphae via myceliogenic germination which can penetrate the sunflower roots, starting its colonization of the host from the base. **(M)** Infection of the roots results in the disease known as basal stalk rot, where the mycelia destroy the root system and work upwards along the stem. **(N)** Infection of the main stem results in the disease, mid stalk rot, which rapidly colonizes and destroys the vascular system, similar to what is seen in soybean and canola. **(O)** Finally, infection of the flower results in sclerotinia head rot of sunflower, where the flower tissues are destroyed, prior to colonization of the stem tissue. Sclerotia formed from all three diseases of sunflower will be deposited into the soil during harvest, where the sclerotia can overwinter and begin its cycle again the following year. Created with BioRender.com.

*S. sclerotiorum* is commonly referred to as a necrotrophic pathogen as it spends the majority of its life cycle necrotizing host tissues. However, recent transcriptomics studies reveal a potentially more nuanced disease progression, demonstrating a marked transition in gene expression following infection and before pathogen-induced host cell death, characteristic of a hemibiotrophic lifestyle (Kabbage et al., 2013). *S. sclerotiorum* overwinters in the soil or within infected debris as either mycelium or sclerotia, which can survive up to 8 years (Adams and Ayers, 1979). The infection cycle for above-ground plant organs is largely similar for soybean and canola, as well as two of the three sunflower diseases, MSR and SHR. Conversely, the disease BSR in sunflower, which begins below the soil surface, exhibits a rather unique disease cycle (**Figure 2**).

In the above-ground diseases, ascospores serve as the primary source of infection in the spring and early summer. Favorable conditions for the carpogenic germination of sclerotia include approximately 10 days of consistent moisture and temperatures ranging from 15-25°C. Apothecia will sprout from the germinated sclerotia following germination, where each apothecium can produce more than 10 million ascospores, for a period of two to three weeks (Agrios, 2005). The spores are then wind disseminated to the host plant, where their germination relies on readily available nutrient sources and moisture. In soybean and canola, successful infection will rely on the spore’s delivery to senescing plant organs, which release their nutrients during senescence. In HR, the sunflower petals have saccharose-producing glands on exterior cells, which is thought to be the main source of nutrients for the germinating ascospore, although senescing petals and pollen may also serve as food sources (Rodríguez et al., 2004). Finally, sunflower MSR is the only one of the above-ground diseases that does not directly involve a host flower. Here, the ascospores will be deposited on the stem or leaf tissue instead. In this case, the nutrients provided for the germination of the ascospore rely on senescing leaves or any pollen that may have fallen from the flower above (Martínez-Force et al., 2015). In all of these scenarios, the acquisition of nutrients by the ascospore provides it with the energy needed to develop an appressorium, which directly penetrates the host tissue allowing the mycelia to begin colonization of the host. The ascospores can also enter the host via natural openings such as stomata, hydathodes, and wounds, although these are less common (Rodríguez et al., 2004; Agrios, 2005; Wang et al., 2015; The Canola Council of Canada, 2020).

Following entrance into the host, the pathogen grows biotrophically and intercellularly for approximately 12–24 hours before undergoing a metabolic transition to a necrotrophic phase (Kabbage et al., 2013). Symptoms in soybean, canola, and sunflower MSR begin subtly with the formation of water-soaked lesions as the hyphae progresses into the stem, releasing numerous effectors and virulence factors into the host tissues. Water-soaked areas quickly transition to pale brown lesions of necrotic tissue that expand radially from the point of infection, eventually girdling the stem, destroying the vascular tissue, and replacing the pith with sclerotia. In these scenarios, the destruction of the vascular tissue will commonly result in wilting, lodging, and eventual death of the plant. In sunflower SHR, the pathogen will proliferate through the florets or the stamens of the flower and will spread to the stem of the plant. Colonization results in water-soaked lesions of the infected flower parts, eventually leading to necrosis and death of the infected tissues. While SHR doesn’t typically result in severe wilting or lodging, infection of the flower head can cause a significant reduction in yield, and large amounts of sclerotia can form in the receptacle of the flower (Bolton et al., 2006).

Alternative to the above-ground diseases is sunflower BSR, where the primary source of infection is mycelia. Basal stalk rot of sunflower is a rather unique disease for *S. sclerotiorum* as sunflower is one of the few hosts that can be colonized myceliogenically through the roots in a natural environment (Bolton et al., 2006). Myceliogenic germination of the sclerotia commonly occurs during periods of moderate temperatures and high relative humidity >80% for a minimum of 12 hours (Saharan and Mehta, 2008). Following germination, the mycelia will colonize the host root tissues via direct penetration of the cuticle, where the pathogen spreads from the roots to the base of the stem, macerating the tissue as it progresses. Maceration of the taproot and lateral roots results in eventual wilting and death of the host, and colonization of the stem base compromises the stem’s integrity, leading to lodging (Lumsden, 1979).

In all of these diseases, secondary infections can occur via mycelia when there is direct contact between healthy and infected plants. In above-ground diseases, dense cropping systems facilitate direct contact of healthy stems with mycelia growing externally on a nearby infected plant. Below ground, in BSR, contact between plant roots is the main cause of the pathogen spreading from one plant to another (Tourneau, 1979).

## 3 Key virulence strategies of *S. sclerotiorum*

**Figure 3:**
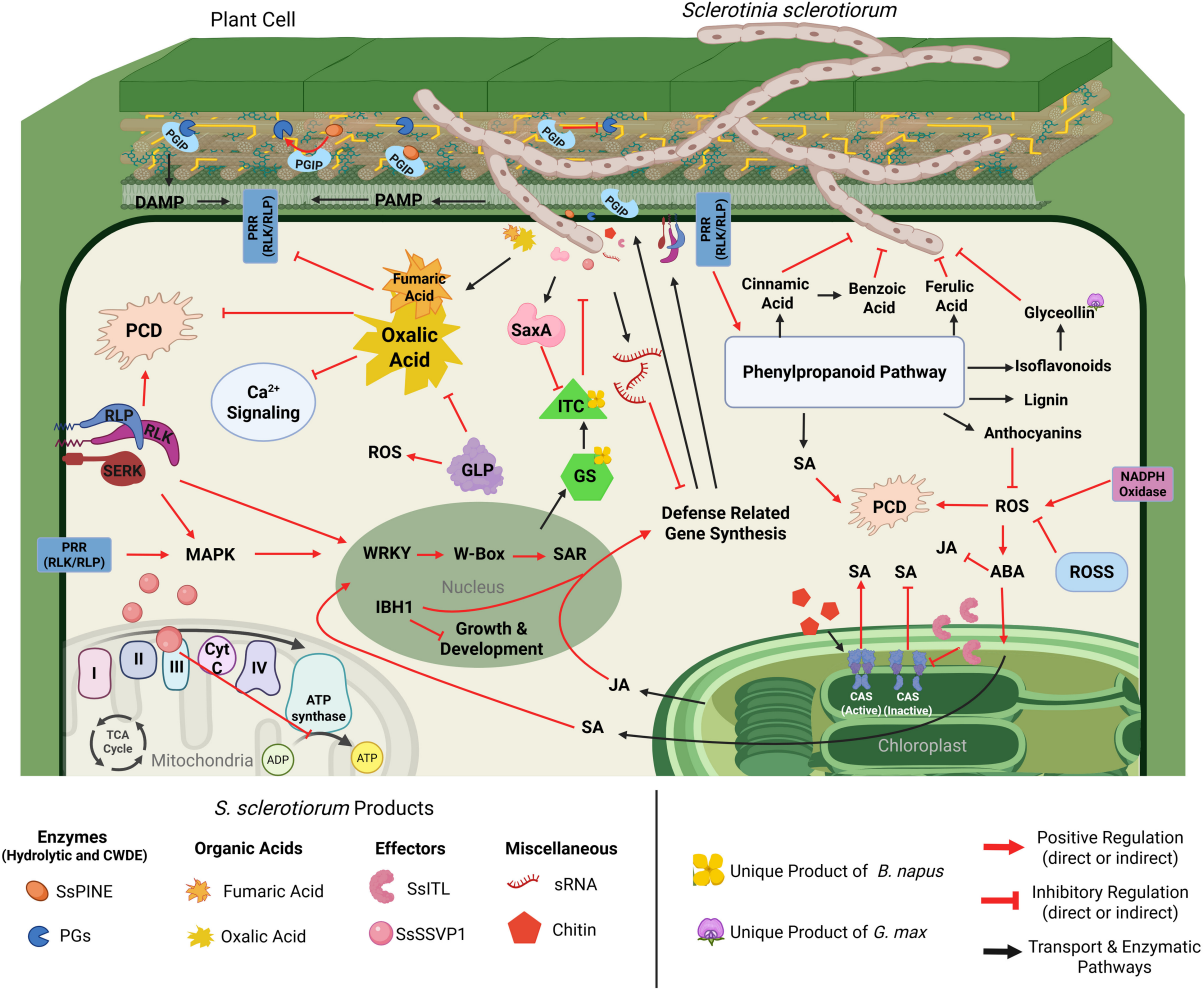
Cellular interaction model detailing *S. sclerotiorum* virulence mechanisms and corresponding defense responses of soybean, canola, and sunflower. Upon penetration of the host cell, *S. sclerotiorum* begins production of plant cell wall degrading enzymes (PCWDEs), including polygalacturonases (PGs), which are responsible for the degradation of the structural cell wall component pectin (yellow lines), resulting in decreased cell stability and production of damage-associated molecular patterns (DAMPs). DAMPs are recognized by host pattern recognition receptors (PRRs), which initiate MAPK signaling cascades that activate transcription factors (TFs) such as WRKYs and IBH1. Activation of TFs results in the synthesis of defense-related genes and hormone signaling. Additionally, certain TFs, such as IBH1, will produce downstream signals to halt the synthesis of genes related to growth and development *S. sclerotiorum*. Defense-related genes such as polygalacturonase inhibiting proteins (PGIPs) are synthesized and shuttled to the cell wall, where they will bind and deactivate PGs. However, *S. sclerotiorum* produces PGIP-inactivating effectors (SsPINE1), which possess a higher binding affinity to PGIP than PGs, resulting in the displacement of bound PGs, furthering the severity of cell wall degradation. Further interference of defense-related gene synthesis can be seen in the production of small RNAs (sRNA) by *S. sclerotiorum*. Some sRNAs produced by *S. sclerotiorum* are complementary to defense-related gene sequences and interfere with their production. Another major product of *S. sclerotiorum* includes oxalic acid and potentially other acids, such as fumaric acid, which inhibit several host defense mechanisms. Deposition of oxalic acid leads to acidification of the host cell, promoting Ca^2+^ chelation, resulting in the interference of Ca^2+^ signaling, dampening host immune responses, and weakening calcium pectate in the middle lamella, assisting in the hydrolysis of pectin via PGs. Furthermore, the suboptimal pH caused by oxalic acid interferes with host protein function and prevents programmed cell death (PCD), further impairing the host’s ability to defend itself. One defense strategy these hosts possess is the production of germin-like proteins (GLPs), which play many roles in the defense against *S. sclerotiorum* infection, but notably, facilitate the production of reactive oxygen species (ROS) and have predicted oxalate oxidase activities, which could help slow the acidification of the host cell. Host cell receptors also initiate defense responses via activation and reprogramming of the phenylpropanoid pathway. The phenylpropanoid pathway produces several defense-related compounds, including cinnamic acid, benzoic acid, and ferulic acid, which have inhibitory activity on *S. sclerotiorum’s* growth and development. Lignin produced from the phenylpropanoid pathway provides structural defense within the cell wall, slowing the incursion of the pathogen. Anthocyanins from the phenylpropanoid pathway have antioxidant properties, which degrade reactive oxygen species, slowing the rate of PCD. Glyceollins, a glycine-specific isoflavonoid (denoted by the soybean flower) are products of the phenylpropanoid pathway, which have been shown to play a vital role in defense against *S. sclerotiorum*, although the exact mechanism is unknown. Finally, the phenylpropanoid pathway is one of the metabolic pathways in plants that produce salicylic acid (SA), which is a critical defense signaling hormone. SA is also produced within the chloroplasts, where changes in calcium concentrations within the thylakoid membrane are detected via host calcium-sensing receptors (CAS). Additionally, CAS activation can occur via the detection of MAMPs such as chitin. CAS activation positively regulates the production of SA in the chloroplasts, improving host defenses. However, *S. sclerotiorum* produces an integrin-like protein (SsITL), which localizes inside of the chloroplast, binding to the CAS proteins and preventing their activation and subsequent mobilization of defense signaling. Further, hormone signaling control is modulated by abscisic acid (ABA), which is understood to be a modulator of plant hormone response. ABA signaling is induced by the production of ROS, where it shifts hormone signals away from growth and development while promoting the further production of SA and antagonizing JA signaling. Another strategy employed by *S. sclerotiorum* is the interruption of host cell energy metabolism via the small secreted virulence-like protein (SsSSVP1), which interacts with the QCR8 subunit of the cytochrome III complex, preventing subcellular localization of the complex and interrupting electron transport and transmembrane proton exchange, inhibiting the production of ATP. A defense-related response specific to plants within the order brassicales (denoted by the canola flower) is the production of glucosinolates (GS). Glucosinolates do not directly inhibit *S. sclerotiorum*; however, the breakdown of GSs results in the formation of isothiocyanates (ITC), which have been shown to inhibit mycelial growth and sclerotial development. In response to the production of ITCs, *S. sclerotiorum* synthesizes enzymes that hydrolyze ITCs (SsSaxA), detoxifying the antifungal metabolites. Created with BioRender.com.

During host invasion, *S. sclerotiorum* has ample strategies to evade host defenses during the early stages of colonization. This is followed by an onslaught of virulence factors, including hydrolytic enzymes, phytotoxic metabolites, and effector proteins (**Figure 3**). All of these factors contribute to the pathogen’s devastating nature (**Table 1**). We will discuss each of these virulence strategies of *S. sclerotiorum* below.

**Table 1:** Summary of known *S. sclerotiorum* virulence factors against the plant hosts, Soybean (Gm), Canola (Bn), and Sunflower (Ha).

Hydrolytic and Cell Wall Degrading Enzymes					
SubGroup	*Gene*/Protein	Host	Host Target	Function	Reference
Polygalacturonases (PG)	PG1/PG1a	Gm, Bn	Pectin in cell walls and primary and middle lamella	Hydrolysis of pectin. Destabilizing host cell and releasing host nutrients for pathogen	(Reymond et al., 1994; Li et al., 2004)
	PG2/PB1b	Gm			(Waksman et al., 1991; Fraissinet-Tachet and Fevre, 1996; Li et al., 2004)
	PG3/PG1c	Gm, Bn			(Waksman et al., 1991; Fraissinet-Tachet and Fevre, 1996; Li et al., 2004)
	PG1d	Bn			(Li et al., 2004)
	PG5	Gm, Bn			(Kasza et al., 2004)
	PG6	Gm, Bn			(Kasza et al., 2004; Li et al., 2004)
	PG7	Gm			(Cotton et al., 2003)
	PGa	Gm			(Favaron et al., 2004)
	PGb	Gm			(Favaron et al., 1992, 2004)
	*Ssxpg1*	Bn			(Li et al., 2004)
	Ssxpg2	Bn			(Li et al., 2004)
PGIP-Inhibitors	SsPINE1	Arabidopsis	PGIP	Inhibit Host PGIP	(Wei et al., 2022b)
Isothiocyanate hydrolase	*SsSaxA*	Bn (crucifers only)		Detoxification of the defense compound isothiocyanate	(Chen et al., 2020)
Organic Acids					
Oxalic Acid	Oxaloacetate acetylhydrolase	Gm, Bn, Ha	Plant cell pH/homeostasis	Oxalic Acid production	(Maxwell, 1973)
Pathogen Effectors					
Proteinaceous Effectors	SsSSVP1	Bn	QCR8 subunit of Cytochrome b-c_1_ complex	Inhibits subcellular localization of the QCR8 subunit within the mitochondria	(Lyu et al., 2016)
	SsITL	Gm, Bn, Ha	Calcium-Sensing Receptor	Prevents normal regulation of SA pathway, dampening immune response	(Zhu et al., 2013)

### 3.1 Hydrolytic and cell wall degrading enzymes

As a predominantly necrotrophic pathogen, *S. sclerotiorum* possesses an array of plant cell wall degrading enzymes (PCWDEs). The largest class of these enzymes are the polygalacturonases (PGs), of which *S. sclerotiorum* has at least 16 (Bolton et al., 2006). PGs are responsible for the degradation of pectin, a critical structural component of plant cell walls, as well as the primary and middle lamella (Kasza et al., 2004). Hydrolysis of structural pectin within the plant cell leads to the decline of cell stability, resulting in the subsequent release of nutrients into the apoplastic environment, which suggests the importance of PGs in cell penetration and fungal proliferation, potentially in the early stages of infection before the necrotrophic phase (Kars and van Kan, 2007). Pectin is a heteropolysaccharide composed of linear chains of D-galacturonic acid residues with various attached side chains (Mohnen, 2008). Wei et al., 2020 identified four *S. sclerotiorum* D-galacturonic acid catabolizing enzymes, namely *Ssgar1*, *Ssgar2*, *Sslgd1*and *Sslga1*, and showed that *Ssgar2*, *Sslgd1*, and *Sslga1* are essential for its virulence on soybean and peas (Wei et al., 2020). In the constant battle between plants and pathogens, plants have evolved a class of cell wall leucine-rich repeat proteins (LRR) known as polygalacturonase inhibiting proteins (PGIPs) (Lorenzo et al., 2001). PGIPs will be discussed in more detail in the plant defense sections of this review. However, PGIPs are effective against PGs in two key ways. First, PGIPs can directly bind to PGs, blocking their ability to degrade cell walls and thereby inhibiting fungal growth. Second, PGs trigger the production of oligosaccharides, which plants can recognize as damage-associated molecular patterns (DAMPs). These DAMPs, in turn, lead to increased production of PGIPs, further elevating the defense response. Incidentally, *S. sclerotiorum* has evolved an extracellular effector capable of inactivating plant defense systems via inhibition of PGIPs (Wei et al., 2022b). Wei and colleagues discovered an *S. sclerotiorum* PGIP-inactivating effector 1 (SsPINE1) that the pathogen utilizes to dampen host immune responses. They determined that SsPINE1 acts as a competitive inhibitor that is preferentially bound by PGIP, allowing it to bind and even replace PG from PGIP after the PG-PGIP complex has formed. The dissociation of PG from PGIP further increases the presence of PGs in the host and significantly contributes to virulence by enhancing PG activity (Wei et al., 2022b). Homologs of SsPINE have been identified in other necrotrophic fungal pathogens such as *Botrytis cinerea*, which suggests ancient evolutionary origins of the gene and sheds light on the timescale of these arms race interactions (Wei et al., 2022b). This impressive adaptation against a highly conserved plant defense mechanism once again demonstrates the sophistication of *S. sclerotiorum* while also lending another potential explanation for its cosmopolitan nature. Another key hydrolytic enzyme at *S. sclerotiorum’s* disposal is isothiocyanate hydrolase (ICTase), an enzyme thought to play a crucial role in the pathogen’s ability to protect itself during infection of plants in the order Brassicales. Isothiocyanates (ITC) are a class of defensive toxins unique to the Brassicales and are synthesized upon herbivory and pathogen attack via glucosinolate activation (Rask et al., 2000; Bednarek et al., 2009). There exists a relative wealth of knowledge on how herbivorous insects circumvent these toxins, including conjugation of hydrolyzed glucosinolate products and, in some cases, by employing symbiotic gut bacteria capable of detoxifying ITC as it is consumed, preventing ill effects in the insect (Ratzka et al., 2002; Wittstock et al., 2004, 2016; Jeschke et al., 2017). ITCs have also been shown to have toxic effects on fungal pathogens, although there is currently very little information regarding fungal detoxification of ITCs (Rahmanpour et al., 2014). Chen et al., 2020 explored the role of glucosinolates and ITCs in host defense against *S. sclerotiorum* as well as the pathogen’s ability to efficiently detoxify ITCs (Chen et al., 2020). They identified and characterized the gene responsible for ITC hydrolysis, naming it *SsSaxA* (*S. sclerotiorum* survival in Arabidopsis extracts) after the hydrolase. They determined from their work that *SsSaxA* can efficiently hydrolyze and detoxify both aliphatic and aromatic forms of ITC, allowing the pathogen to circumvent the antifungal metabolites produced during the plant defense reaction.

Additionally, they observed that ITC produced by the plants began accumulating as soon as 6 hours post inoculation (hpi) and reached a peak at 24hpi, followed by a substantial decrease at 48hpi, which they attributed to *SsSaxA* activity as the drop in ITC levels coincided with an increase in hydrolyzed ITC products within the leaves. Further work is needed to confirm the peak expression timing of this hydrolytic enzyme. However, this study reveals *SsSaxA* as a critical player during the later stages of the infection process (Chen et al., 2020).

### 3.2 Organic acids

The non-specific phytotoxin oxalic acid (OA) is arguably the most well-studied and commonly discussed metabolite of *S. sclerotiorum*. Oxalic acid’s contribution to *S. sclerotiorum’s* pathogenicity is a direct result of the acidification of the host cell (Dutton and Evans, 1996; Palmieri et al., 2019). Suboptimal pH within the plant cell promotes the chelation of Ca^2+^, which interferes with calcium signaling and chelates calcium pectate within the middle lamella, facilitating the hydrolysis of pectate via pathogen polygalacturonases (Magro et al., 1984; Dutton and Evans, 1996; Monazzah et al., 2018a). There exists a debate on whether OA should be considered a virulence factor or simply a modulator of host physiology (Callahan and Rowe, 1991). Regardless of exact classification, it is clear that the disruption of host cell homeostasis imparted by the release of OA results in the loss of a plant’s ability to defend itself effectively, increasing the pathogen’s virulence. The role of oxalic acid in the virulence of *S. sclerotiorum* has been covered extensively in the past, and detailed reviews have been completed by Xu et al. in the Annual Review of Phytopathology in 2018 and Gokul et al. in the Physiological and Molecular Plant Pathology Journal in 2024 (Xu et al., 2018; Daniel et al., 2024).

Continuing the investigation into host cell acidification and its impact on virulence, Xu et al., in 2015, produced mutants defective in oxaloacetate acetylhydrolase (OAH), the enzyme responsible for OA production in *S. sclerotiorum* (Xu et al., 2015)). Their study discovered that *oah* mutants lost their ability to accumulate OA during infection. However, contrary to their hypothesis that OA is *S. sclerotiorum’s* sole virulence factor, these mutants retained their ability to induce symptoms in Faba bean and pea but almost entirely lost their virulence in soybean. The proposed explanation for this difference was that soybeans, compared to the other hosts in this experiment, have the highest pH and buffering capacity, thus disallowing *S. sclerotiorum* from properly modulating its environment. This observed maintenance of virulence in specific hosts coincided with an accumulation of fumaric acid. They demonstrated that fumaric acid, a relatively weaker acid, accumulated in infected leaves to such an extent that the tissue pH reached nearly that of leaves infected by WT strains. This finding does not directly disprove the importance of OA; instead, it further demonstrates the pathogen’s aggressiveness and adaptability. While *S. sclerotiorum* clearly displays a preference for the production of OA in establishing a conducive environment for infection, it seems fully capable of producing fumaric acid as an alternate virulence factor. This adaptability could further explain this pathogen’s lack of host preference.

### 3.3 Proteinaceous effectors

In nature, pathogens secrete effectors, most often as proteins, to infect and survive in the host plants (Giraldo et al., 2013). These effectors are utilized by pathogens to manipulate host cellular processes to their advantage. The list of effector proteins in *S. sclerotiorum’s* arsenal is robust and ever-growing. A recent study that produced an updated version of the complete genome sequence of *S. sclerotiorum* reported a total of 70 putative effectors via EffectorP analysis (Derbyshire et al., 2017). Pathogen effectors have many functions throughout the disease cycle, ranging from evading host detection, dampening immune responses, and hijacking biochemical pathways for their benefit (Rovenich et al., 2014). A large majority of the proteinaceous effectors described for *S. sclerotiorum* are involved in disrupting immune signaling and induction of host cell death. One such effector protein described by Lyu et al. is the Small Secreted Virulence-like Protein 1 (SsSSVP1). SsSSVP1 was found to be highly expressed as soon as 3 hours post-inoculation (hpi) and reached its highest expression level at 12hpi, inducing rapid cell death following its translocation into the host cell (Lyu et al., 2016). SsSSVP1 interacts with a QCR8 subunit of the cytochrome b-c_1_ complex, inhibiting subcellular localization within the mitochondria. The authors suggest that small secretory protein has two distinct yet compounding effects during infection. The first, as a toxin, leads directly to rapid cell death. The second, as an impediment to plant energy metabolism, results in the gradual weakening of the cell and, eventually, cell death. Because this protein is expressed early in the infection process, it could potentially serve to exhaust the plant’s defense machinery before *S. sclerotiorum* releases its other effectors, increasing its virulence and resulting in a more rapid collapse of the plant upon its transition to necrotrophy.

Another major secretory protein employed by *S. sclerotiorum* is the integrin-like protein SsITL, first described by Zhu et al., 2013. While SsITL contains homology to that of other integrin proteins, it does not contain a transmembrane domain and was determined to function as a secretory protein that localizes within the host cells during infection. Expression analysis indicated that SsITL is highly expressed 90 minutes following infection and displayed peak expression levels at 3hpi. Initial findings demonstrated that SsITL suppressed host immune responses very early in the disease cycle via an interruption of jasmonic acid (JA) and ethylene (Et) signaling, prevention of salicylic acid (SA) accumulation, and interference of crosstalk between the two antagonistic pathways. However, the mechanism for this interference was unknown until 2020, when Tang et al. discovered that SsITL interacts with a calcium-sensing receptor (CAS), which functions as a positive regulator of SA signaling and, therefore, plant immune responses (Tang et al., 2020). The CAS receptor recognizes and responds to the presence of the microbe-associated molecular pattern (MAMP) chitin, rapidly promoting the expression of SA biosynthesis genes (Nomura et al., 2012). Rapid accumulation of SsITL during early infection allows *S. sclerotiorum* to immediately disarm the plant’s ability to sense and respond to the invasion. These examples show *S. sclerotiorum* possesses many robust strategies for suppressing host immune responses. Moreover, a common theme emerges, demonstrating that the first organism to react in the host-pathogen interaction will likely prevail.

### 3.4 Small RNAs

Small RNAs (sRNA) are RNA molecules ranging from 20 to 30 nucleotides in length and are non-coding (Dang et al., 2011). While sRNAs do not directly code for genes, they are known to play a crucial role in endogenous gene expression via RNA interference (RNAi). The binding of complementary sequences by sRNAs results in the silencing or repression of gene expression, which is crucial for growth and development, as well as response to the environment (Baulcombe, 2015). In addition to endogenous interactions, sRNAs secreted by fungal pathogens into host tissues can result in targeted host gene silencing, impeding host defense mechanisms and facilitating infection (Weiberg et al., 2013). In this sense, sRNAs can function similarly to pathogen-effector proteins. Yet more work needs to be done to discover sRNAs of *S. sclerotiorum.* In 2019, Derbyshire et al. conducted the first known study into the production of sRNA by *S. sclerotiorum* during plant infection (Derbyshire et al., 2019). This study revealed 374 sRNA sequences that were significantly upregulated during the infection of both *Arabidopsis thaliana* and *Phaseolus vulgaris.* Furthermore, the predicted targets of these sRNAs in *A. thaliana* were involved in host disease resistance. This was tested by silencing two of these predicted targets, *SERK2* and *SNAK2* in *A. thaliana*, resulting in increased susceptibility in the mutant lines. This work clearly demonstrates the need for further study into the utilization of sRNA by *S. sclerotiorum*, especially in other important oil seed hosts, to better understand and defend against the pathogenic mechanisms of *S. sclerotiorum.*

## 4 Defense mechanism of soybean to *S. sclerotiorum*

In soybean, *S. sclerotiorum* causes stem rot known as Sclerotinia stem rot (SSR). SSR consistently ranks among the top yield-limiting soybean diseases in the continental United States. In 2021, SSR ranked first as the most devastating fungal disease of soybean in the U.S.A., resulting in a loss of more than 25.7 million bushels, totaling nearly $335,000,000, and ranked as the second most important yield-limiting soybean disease (Crop Protection Network, 2023).

For an effective defense response, the most crucial time during soybean infection is within the first 12 hours following germination of the ascospore (Rothmann and McLaren, 2018). Partially resistant soybean varieties show significant and rapid changes in gene expression and metabolite accumulation following infection compared to susceptible varieties (Kabbage et al., 2013; Ranjan et al., 2019; Zhang et al., 2022b). This observation suggests that the timing of the response might be more impactful than the quantity or quality of the response, although that has yet to be directly studied. Furthermore, the transition of *S. sclerotiorum’s* ephemeral biotrophic phase to a necrotrophic lifestyle places even greater importance on a plant’s ability to recognize the threat, respond quickly, and shift rapidly from a biotrophic defense strategy to one that defends more effectively against necrotrophic pathogens. In studying this transition, Kabbage et al. reported that during the biotrophic phase, *Arabidopsis* plants initiated a hypersensitive (HR) response, typical of a biotrophic pathogen response, resulting in the initiation of programmed cell death (PCD) meant to quarantine the pathogen (Kabbage et al., 2013). However, upon transition to necrotrophy, the pathogen leverages this response to procure nutrients from these cells, fortifying its necrotizing abilities.

Studies conducted on quantitative trait loci (QTL) are a powerful tool in understanding pathogen-host interactions for uncovering the mechanisms behind disease resistance. Numerous QTL studies have been conducted concerning SSR and soybean interactions. In 2010, Li et al. identified three QTLs in the relatively tolerant soybean line Maple Arrow, associated with soluble pigment content and SSR resistance (Li et al., 2010). Reports prior to this study have mainly focused on QTLs that are related to disease escape mechanisms, including flowering date, canopy architecture, and maturity groups, leading Li et al. to focus on QTLs associated with resistance rather than escape mechanism phenotypes, which can be agronomically useful in various systems but do not directly relate to resistance response (Kim et al., 1999). The utility of identifying QTLs associated with soluble pigment content lies in secondary metabolite and anthocyanin production in response to pathogen attack (Li et al., 2010). A follow-up study exploring the soluble pigment content of soybean stems identified a QTL named Qswm13-1, located on chromosome 13, was found to explain 23.62% of stem pigment variation, which the authors believed to be related to candidate genes beneficial to resistance response (Zhao et al., 2015). Future studies should focus on the characterization of candidate genes associated with this marker for utilization in breeding programs and engineering for improved resistance.

While no completely resistant soybean varieties have been identified, these plants still have several defense strategies at their disposal when facing *S. sclerotiorum* infection (**Table 2**). These responses include the production of phytoalexins, intermediary metabolites, pathogenesis-related proteins (PRRs), reactive oxygen species (ROS) scavengers, and countless transcription factors (**Figure 3**). These plant defense strategies have been discussed below in detail.

**Table 2:** Summary of the plant hosts soybean (Gm), canola (Bn), and Sunflower (Ha) defense strategies against *Sclerotinia sclerotiorum*.

Defense Strategy	Plant Host	Gene/Protein	Product/Role	Citation
Phenylpropanoid Intermediates	Gm, Bn, Ha	*GmPAL* *BnPAL* *HaPAL*	Cinnamic Acid	(Liu et al., 2017; Monazzah et al., 2018a; Ranjan et al., 2019; Zhang et al., 2023)
	Gm, Bn, Ha	Several Enzymatic Steps	Benzoic Acid	(Barz et al., 1978; Naczk and Shahidi, 1992; Honiges et al., 2017)
	Gm, Bn, Ha	*GmCOMT* *BnCOMT* *HaCOMT*	Ferulic Acid	(Cabello-Hurtado et al., 1998; dos Santos et al., 2004; Bhinu et al., 2009; Badouin et al., 2017)
	Gm, Bn, Ha	*GmCCoAOMT* *BnCCoAOMT* *HaCCoAOMT*	Monolignol Precursors	(Badouin et al., 2017; Ding et al., 2021; Guo et al., 2023)
	Gm	Glyceollin synthase (GS)	Glyceollin I, II, III	(Banks and Dewick, 1983; Anguraj Vadivel et al., 2016; Wang et al., 2018a)
Polygalacturonase Inhibiting Proteins (PGIP)	Gm, Bn, Ha	GmPGIP1-11 BnPGIP1-17 HaPGIP1-4	Defense against fungal PGs	(D’Ovidio et al., 2006; Hegedus et al., 2008; Kalunke et al., 2014; Livaja et al., 2016; Acharya et al., 2024)
ROS Production	Gm, Bn	NADPH Oxidases/RBOH	O_2_^-^ Production	(Fan et al., 2014; Ranjan et al., 2018; Wang et al., 2018b; Liu et al., 2019)
ROS Scavenging	Gm, Bn, Ha	Peroxidases	Antioxidant	(Sessa and Anderson, 1981; Dalton et al., 1986, 2009; Schenk et al., 2008; Gopavajhula et al., 2013)
	Gm, Bn, Ha	Glutathione S-transferases	Antioxidant	(Dalton et al., 1986, 2009; Ma et al., 2018; Zhang et al., 2023)
	Gm, Bn, Ha	Superoxide dismutases	Antioxidant	(Fernández-Ocaña et al., 2011; Gopavajhula et al., 2013; Su et al., 2021)
	Gm, Ha	Ascorbate oxidases	Antioxidant	(Dalton et al., 1986; Zhang and Kirkham, 1996)
	Bn	Ascorbate peroxidase	Antioxidant	(Nováková et al., 2014; Liu et al., 2017; Monazzah et al., 2018b; Ranjan et al., 2019; Wei et al., 2022a; Rai et al., 2024)
Transcription Factors	Gm, Bn, Ha	bZIP	Regulates ROS Scavenging Genes	(Zhou et al., 2017; Zhang et al., 2021; Rahman et al., 2023; Xiao et al., 2023)
	Gm, Bn, Ha	GmWRKY33 BnWRKY33 HaWRKY7 (WRKY33 Homolog)	Directs expression of various defense responses	(Zheng et al., 2006; Giacomelli et al., 2010; Liu et al., 2018; Xiao et al., 2023)
Phytohormones	Gm, Bn, Ha	SA/JA/ABA	Defense Signaling	(Nováková et al., 2014; Liu et al., 2017; Monazzah et al., 2018b; Ranjan et al., 2019; Wei et al., 2022a; Rai et al., 2024)
Glucosinolates	Bn	Isothiocyanates	Antifungal Properties inhibiting sclerotial germination and mycelial growth	For full table, please refer to (Plaszkó et al., 2021)
PR Proteins	Ha	HaPR5	Disruption of fungal β-glucans and modulation of plant defense response	(Alignan et al., 2006)
	Ha	HaGLP1	Increased ROS Production	(Fernández et al., 2003; Beracochea et al., 2015)

### 4.1 Secondary metabolites (phenylpropanoid products and lignin intermediates)

Plant-derived secondary metabolites play a crucial role in defending against microbial pathogens. One of the major plant secondary metabolite biosynthesis pathways is the phenylpropanoid biosynthesis pathway. The phenylpropanoid biosynthesis pathway produces several key metabolites, such as flavonoids, phytoalexins, and lignin precursors, which have also shown promise in the search for improved pathogen resistance. In a transcriptomics and metabolomics study comparing resistant and susceptible responses to SSR infection, resistant lines produced increased levels of cinnamic acid, benzoic acid, and ferulic acid. In resistant lines, metabolite production nearly doubled for cinnamic acid as soon as 6hpi. In contrast, ferulic acid had doubled by 72 hpi, and benzoic acid displayed an impressive 6-fold increase at 72 hpi (Ranjan et al., 2019). Furthermore, all three intermediates inhibited *S. sclerotiorum* growth in amended agarose plates, demonstrating their antifungal properties (Ranjan et al., 2019; Wang et al., 2019). The timing and production of these antimicrobial intermediates are of great interest, as cinnamic acid is immediately upstream of benzoic acid in the phenylpropanoid biosynthesis pathway. However, their peak heights are 66 hours apart, potentially implicating cinnamic acid’s role during early infection against biotrophic pathogens, whereas, at 72 hpi, *S. sclerotiorum’s* necrotrophic machinery is in complete control (Ranjan et al., 2019).

In addition to these antimicrobial metabolites, soybeans produce many vital enzymes that facilitate defense responses. Of these, S-adenosyl-L-methionine-dependent methyltransferases superfamily proteins (SAM-Mtases), also known as caffeoyl coenzyme A O-methyltransferases (CCoAOMT), and calcium-binding proteins have all shown significant association with *S. sclerotiorum* disease response, via QTL analysis (Wang et al., 2015; Zhang et al., 2022b). The CCoAOMT gene family is positioned within the phenylpropanoid pathway and catalyzes the conversion of caffeoyl-CoA to feruloyl-CoA by adding an O-methoxyl group to the 3’ position of the aromatic ring (Vanholme et al., 2010). Feruloyl-CoA’s production directs the production of various aldehydes, monolignols, and eventually syringyl and guaiacyl lignin (Kim et al., 2010a); Ranjan et al., 2019). Lignin and aldehyde intermediates have shown great importance in plant structure and defense. However, the *GmCCoAOMT* gene family has not been fully characterized, and work in this area may shed light on novel strategies for improving SSR resistance in soybean. Phytoalexins are a broad class of toxic antimicrobial compounds produced by plants in response to pathogen infection. In legumes, isoflavonoids comprise the majority of phytoalexin products and are produced as secondary metabolites from the phenylpropanoid pathway. Among these isoflavonoids exists a class of *Glycine-*specific metabolites known as glyceollins, which are synthesized from the isoflavonoid branch of the phenylpropanoid pathway (Banks and Dewick, 1983; Lygin et al., 2010; Anguraj Vadivel et al., 2016). Glyceollin production was upregulated in response to a range of pathogens, demonstrating its relation to soybean defense response. Moreover, *S. sclerotiorum* and five other fungal pathogens of soybean all displayed growth inhibition when grown in cultures amended with glyceollin (Darvill and Albersheim, 1984; Sharma and Salunkhe, 1991; Lygin et al., 2010). However, in the same study, Lygin et al. found that *S. sclerotiorum* could metabolize glyceollin in culture, reporting that less than 2% of the glyceollin added to the plate remained once fully colonized, further exemplifying the devastating nature and impressive adaptations of this fungal pathogen. While the exact mode of inhibition is unknown, it is clear that glyceollins play a vital role in defense response. Therefore, further study of its specific function and mode of action could reveal critical insights into developing durable resistance.

### 4.2 Polygalacturonase-inhibiting proteins

Another group of key defense molecules produced by soybean is a group of membrane proteins known as polygalacturonase-inhibiting proteins (PGIPs) (Kalunke et al., 2015). Many fungal pathogens, including *S. sclerotiorum*, produce PGs along with other pectin-degrading enzymes during pathogenesis, contributing to their virulence and facilitating nutrient acquisition. To defend themselves, soybeans produce PGIPs, which are members of the leucine-rich repeat (LRRs) glycoproteins commonly found in the plant cell wall (Lorenzo et al., 2001). To date, 11 *PGIP* genes have been identified in *G. max* which are spread across four chromosomes. Four copies are located on chromosomes 5 and 8, two on chromosome 15 and one on chromosome 19 (Acharya et al., 2024). The multiple *PGIPs* found on chromosomes 5, 8, and 15 are likely due to the two gene duplication events (D’Ovidio et al., 2006; Kalunke et al., 2014; Acharya et al., 2024). Kalunke et al. expanded the list of *GmPGIPs* from four to six in 2014, where they performed sequencing and transcriptomics experiments to identify and characterize the expression changes of the six PGIPs in response to *S. sclerotiorum* infection. As a group of resistance-related proteins, it is unsurprising that they observed increased expression levels of the PGIP genes following infection. Moreover, they showed that the different genes showed peak expression increases at various times, demonstrating that they may function independently or at least distinctly against the variety of PGs used by the pathogen. Importantly, they observed that the expression level of *GmPGIP5* steadily decreased from 8 hpi to 24 hpi, followed by a 1000-fold increase from 24 hpi to 48 hpi. In *GmPGIP7*, expression levels remained low yet steadily increased from 8 hpi to 24 hpi, with a 30-fold increase from 24 hpi to 48 hpi. While this gene family is considered to be crucial for resistance against necrotrophic fungal pathogens, specifically containing PGs, the authors report that by the time the expression levels rose, around the 48-hour mark, the plant tissues were already displaying necrosis and maceration by *S. sclerotiorum*, indicating that the response may have been too late to be fully effective (Kalunke et al., 2014). These results are consistent with the hemibiotrophic model of *S. sclerotiorum’s* lifestyle as well as the idea that response timing may be a more critical factor than the type of response or possession of proper machinery. While not explored in the aforementioned study, it is not hard to imagine that *S. sclerotiorum* evolved an array of effectors, which during early biotrophic stages of infection, signal to the plant that it is facing a biotrophic pathogen. This trojan horse-style strategy could explain the decrease in *PGIP5* expression in the first 24 hours, followed by the rapid and drastic increase in *PGIP5* and *PGIP7* expression at 48 hours once the pathogen has transitioned to the necrotrophic stage. Moreover, once the pathogen begins producing and releasing its CWDEs, PGs, and effectors around 24 hpi, the plant clearly responds with a significant increase in defense gene expression; however, these results demonstrate that the response, while bountiful, was too late to confer any meaningful protection.

While this research on PGIP expression and gene duplication is important, no further studies have been conducted on the relationship between soybean defense against *S. sclerotiorum* and PGIPs, highlighting the lack of current knowledge in an area that is likely to hold a repository of crucial information about the improvement of resistance responses to SSR.

### 4.3 Reactive oxygen species

Reactive oxygen species (ROS) production is ubiquitous in the plant kingdom and is produced at relatively lower levels during normal cellular metabolism (Scandalios et al., 1997). While plants possess scavenging mechanisms to degrade these basal levels of ROS, they are known to be highly overproduced during a stress response, reaching toxic levels (Kotchoni and Gachomo, 2006; Mittler et al., 2011; Noctor et al., 2014; Xia et al., 2015; Sewelam et al., 2016). The toxic accumulation of ROS results in localized cell death, a typical plant response for quarantining and defending against biotrophic pathogens (Ranjan et al., 2018). However, programmed cell death via ROS bursts may facilitate infection of necrotrophic pathogens, such as *S. sclerotiorum*. ROS in plants can come from several different sources, but one of the major contributors includes the membrane-bound NADPH oxidases, which catalyze the conversion of O_2_ to O_2_^-^ (Sagi and Fluhr, 2001). In 2018, Ranjan et al. identified and characterized four respiratory burst oxidase homologs of soybean (GmRBOH) that are significantly upregulated upon *S. sclerotiorum* infection, confirming their role in the infection process. Additionally, they found that virus-induced gene silencing (VIGS) of this group of *GmRBOH* genes resulted in increased resistance to the fungal pathogen (Ranjan et al., 2018). These results suggest that ROS production via NADPH oxidases may aggravate host infection by facilitating the necrotrophic phase of this fungal pathogen.

However, plants possess several mechanisms to prevent the overaccumulation of various oxidative stressors with the use of antioxidant scavenging molecules. The ability to modulate ROS accumulation via scavenging improves the plants’ ability to fine-tune its response to biotic and abiotic stresses that commonly trigger ROS bursts. In soybean, genes related to ROS scavenging include peroxidases, glutathione S-transferases (GST), ascorbate oxidases, and superoxide dismutases, to name a few (Sessa and Anderson, 1981; Dalton et al., 1986, 2009; Gopavajhula et al., 2013). Additionally, anthocyanins have long been recognized for their antioxidant capacity in plants. Ranjan et al. explored the differential expression of these genes in response to *S. sclerotiorum* infection in resistant and susceptible lines. They found that resistant soybean lines showed significantly different expression of these genes during defense response compared to susceptible plants. They identified five GSTs, five peroxidases, five ascorbate oxidases, and two superoxide dismutases that showed significant upregulation between 24-96hpi, demonstrating the ability of the resistant lines to efficiently respond and detoxify the ROS accumulation related to SSR infection. Regarding anthocyanins, they found five of seven anthocyanin-related genes to be upregulated following expression, importantly, most reaching their peak expression levels at 96hpi. This evidence provides another line of reasoning pointing to the hijacking of ROS accumulation by *S. sclerotiorum* at later stages of infection while demonstrating the resistant lines’ ability to recognize and respond to these accumulations. Further work should be completed to explore how to increase the antioxidant capacity of soybean and other hosts in defense against *S. sclerotiorum* attack (Ranjan et al., 2019).

### 4.4 Transcription factors

Transcription factors (TFs) are class of proteins regulating genes’ transcript levels (Latchman, 1997). Transcription factors related to plant-pathogen defense are activated in response to biotic stimuli, resulting in effective signal transduction and cellular communication within the plant (Schluttenhofer and Yuan, 2015; Zhang et al., 2018). In 2021, Zhang et al. characterized the soybean bZIP transcription factor *GmbZIP15* in its role in *S. sclerotiorum* defense response. They found that upregulation of *GmbZIP15* increased the resistance response to *S. sclerotiorum* in soybean by increasing the transcription of ROS scavenging genes, allowing the plant to better defend against RBOH responses to SSR. Furthermore, they confirmed that introducing phytohormones, such as SA, JA, and ethylene, induced the function of this bZIP TF (Zhang et al., 2021). In a separate study, Xiao et al. explored the role of *GmSWEET* genes in *S. sclerotiorum* response. SWEET transporters are known for their role in carbohydrate transport across cell membranes. These transport genes have been extensively studied in other plant-pathogen interactions, namely those between rice and *Xanthomonas oryzae* pv. *oryzae*; however, their role in soybean-*Sclerotinia* interactions had not previously been reported. They observed that *gmsweet15* mutants had reduced lesion area and pathogen colonization compared to WT lines, which they explained to be the result of drastic transcriptional reprogramming due to the mutation. Specifically, they found that transcription factors such as *WRKY33* and heat stress transcription factors (*HSF5, HSF36*) were positively correlated with the phenotype observed in *gmsweet15* mutants. In contrast, bZIP and MYB transcription factors were negatively correlated with the mutant phenotype (Xiao et al., 2023). As we progress our knowledge of the many defense-related interactions between soybean and *Sclerotinia*, studies like these add to our list of potential quantitative factors related to disease resistance in this pathosystem while also highlighting the vast number of interactions that remain unexplored.

### 4.5 Phytohormones

Phytohormones facilitate a plant’s ability to respond to both biotic and abiotic changes to its environment (Bari and Jones, 2009; Denancé et al., 2013). Key phytohormones commonly associated with defense signaling in response to pathogens include, but are not limited to salicylic acid (SA), jasmonic acid (JA), and abscisic acid (ABA) (Jones and Dangl, 2006; Zheng et al., 2006; Bari and Jones, 2009). In a 2022 study, Wie et al. found that upon infection by *S. sclerotiorum* gene-ontology terms for JA were enriched in both resistant and susceptible genotypes as soon as 8 hours post infection (Wei et al., 2022a). In another study by Ranjan et al. in 2019, it was found that the phytohormones salicylic acid (SA), jasmonic acid (JA), and abscisic acid (ABA) were differentially utilized in defense response when comparing resistant and susceptible lines, further implicating the importance of hormone signaling in pathogen defense. In a metabolite analysis study conducted by Ranjan et al., they report several findings related to the quantity and timing of phytohormone response, looking at hormone production dynamics at 6, 12, 24, 48, and 72hpi. Beginning with SA, they observed that susceptible lines produced nearly a two-fold increase in SA production across all time points when compared to the resistant variety, which, in comparison, showed a steady decrease in SA production. When assessing JA production, the resistant and susceptible lines were indistinguishable until 48 and 72hpi, where the susceptible line shifted from around 200 pmoles/g of tissue to nearly 800 pmole/g, while the resistant line dropped to nearly zero by 72hpi. Finally, ABA showed a significant increase in production in the susceptible line at 12hpi; however, the resistant line began producing more ABA at both 48hpi and 72hpi. As an overall modulator of plant hormone response, it can be understood that the peak in ABA in the resistant line took control at the later time points, antagonizing and reducing the JA signal (Ranjan et al., 2019). Future research should focus on the precise mechanisms controlling the timing and quantity of phytohormone signaling to better improve the defense response reactions in the presence of pathogen attack.

## 5 Defense mechanisms of canola to *S. sclerotiorum*

Sclerotinia stem rot (SSR) of canola is often referred to as the most destructive threat to canola production in Canada, where disease prevalence of up to 79% has been reported (The Canola Council of Canada, 2020). *B. napus* has several defense mechanisms that can be used in defending against SSR, and some relatively tolerant canola varieties have been identified in wild relatives such as *B. oleracea* (Mei et al., 2015; Taylor et al., 2018). Still, as in other hosts, complete resistance has yet to be found. Of the defense mechanisms that canola can employ, it has a range of inducible phytoalexins, including flavonoids and terpenoids, which possess antimicrobial activity (**Figure 3**). Additionally, canola can produce key metabolites such as glucosinolates and brassinosteroids, which are unique to the brassica family (**Table 2**).

Other defense strategies of canola that are seldom discussed in soybean and sunflower include various avoidance strategies. When the ascospore begins colonization of the senescing flower, the flower may be shed before the pathogen reaches the stem. Additionally, if a lateral branch is infected, the plant may abscise the branch, successfully stopping the pathogen from reaching the main stem and avoiding the death of the whole plant. However, these detached flowers and stems may land on a leaf below them or a neighboring plant, which would, again, result in further infection (Wang et al., 2015). Understanding how canola can sense and respond to the oncoming invasion may provide useful insights into developing plants that have improved avoidance strategies.

### 5.1 Glucosinolates

Glucosinolates are a class of organic compounds derived from glucose and amino acid precursors. Glucosinolates do not possess direct inhibitory properties by themselves but instead are enzymatically altered within the plant to produce bioactive defense compounds (Halkier and Du, 1997; Wittstock et al., 2016). The most well-studied inhibitory product from glucosinolate breakdown is isothiocyanate (ITC), which was reviewed thoroughly by (Plaszkó et al., 2021). However, there are a few key points worth mentioning. These antimicrobial compounds have been shown to inhibit mycelial growth and sclerotial germination of *S. sclerotiorum*, demonstrating their importance in the defense response to SSR infection (Manici et al., 1997; Kurt et al., 2011; Dhingra et al., 2013; Sotelo et al., 2015). Although, Rahmanpour et al. found that repeated exposures to ITC lead to the pathogen developing significant tolerance to high doses of ITC, exemplifying the difficulties in combating this pathogen (Rahmanpour et al., 2009). However, glucosinolates possess significant variation in their side chain chemistry, resulting in a multitude of degradation products that can be produced in varying quantities (Plaszkó et al., 2021). These studies have focused on one or a few variations of ITCs individually, and further work should focus on investigating the many permutations of ITC combinations and concentrations, as it is unlikely that *S. sclerotiorum* could recognize and detoxify all of them simultaneously.

### 5.2 Polygalacturonase-inhibiting proteins

Similar to soybean, canola utilizes PGIPs to defend against pathogen attack by inactivating fungal PGs (Li et al., 2004; Hegedus et al., 2008). At the time of writing, 17 BnPGIPs have been identified, having differential responses to various biotic and abiotic stimuli (Hegedus et al., 2008; Wang et al., 2021). Given the range of stimuli PGIPs can respond to, Hegedus et al. sought to identify which *BnPGIPs* were induced during *S. sclerotiorum* infection. Their results indicated that the 16 BnPGIPs clustered into three groups that were named BnPGIP1, BnPGIP2, or intermediate, based on sequence similarity. Among these groups, they found that the BnPGIP2 group, consisting of *BnPGIP2, 5, 6, 9, 12*, and *16*, as well as *BnPGIP8* (intermediate), all showed increased expression levels following *S. sclerotiorum* infection (Hegedus et al., 2008). Given the range of BnPGIPs and SsPGs, Wang et al. assessed the interactions between BnPGIPs and SsPGs. Additionally, they performed functional studies assessing the expression levels and overexpression phenotypes of *BnPGIP2, 5*, and *10* in relation to *S. sclerotiorum* infection. First, they determined that *BnPGIP2* and *10* were greatly upregulated in leaf tissues following *S. sclerotiorum* infection, while *BnPGIP5* only showed slight increases in expression levels compared to non-infected tissues. Next, in assessing the disease responses of canola lines overexpressing each of the three *PGIPs* they observed that *BnPGIP2-OE* lines developed significantly smaller lesions on both stem and leaf tissues, confirming *BnPGIP2’s* role in an improved defense response. Perhaps unexpectedly, *BnPGIP5-OE* lines also had significant reductions in lesion size, where *BnPGIP10-OE* was not different from the wild type at any time point or tissue type. Finally, in determining which BnPGIPs interact with SsPGs they found that BnPGIP2 and BnPGIP5, but not BnPGIP10, directly inhibit the function of SsPG crude extracts, proving their importance in defense against *S. sclerotiorum* infection (Wang et al., 2021). These studies highlight the importance of performing functional characterization to determine the role and interactions of host and pathogen proteins and further exemplify the nuances that exist within these systems.

### 5.3 Mitogen-activated protein kinases

Mitogen-activated protein kinases (MAPKs) are well known in the plant kingdom for their role in innate plant immunity responses (Jones and Dangl, 2006). MAPK cascades are mediated by effector-triggered immunity (ETI), and pathogen-associated molecular pattern (PAMP) triggered immunity (PTI), where a cascade of phosphorylation events converts MAPKKK to MAPKK to MAPK, eventually leading to the phosphorylation and subsequent activation of defense-related transcription factors (Bigeard et al., 2015; Cui et al., 2015). While this immune response pathway is generally recognized, its role in *B. napus* response to *S. sclerotiorum* was poorly understood until relatively recently. In 2022, Zhang et al. identified and characterized the BnaA03.MKK5 and BnaA06.MPK3/BnaC03.MPK3, determining their roles in positive modulation in response to SSR. It was determined that MAPKK peaks at 12hpi, followed by a MAPK peak at 24hpi, confirming this phosphorylation cascade and its importance in plant-pathogen defense in this pathosystem. Additionally, they found that overexpression of BnaA03.MKK5 delayed lesion expansion compared to WT lines when infected with *S. sclerotiorum* (Zhang et al., 2022b).

### 5.4 Transcription factors

WRKY transcription factors are key players in many plant-microbe interactions that regulate the transcription levels of various defense and stress-related genes, allowing plants to respond appropriately to a wide range of elicitors. WRKYs are proteins that contain a DNA binding domain consisting of a conserved WRKYGQK motif at their N-terminus and a zinc-binding motif at their C-terminus. This N-terminus DNA binding domain interacts with W-box cis-element in the promoter region of targeted defense-related genes to activate or inhibit their transcription (Dong et al., 2024). The WRKY family includes several types of WRKY proteins, the largest group being transcriptional regulators, commonly associated with response to biotic and abiotic stimuli. Additionally, WRKYs facilitate the regulation of pathogenesis-related gene expression. Regulation of WRKY genes is controlled by phosphorylation events via MAPK cascades, protein-protein interaction, and protein degradation via the proteasome, which limits the duration of activation or repression of genes by transcription factors (Dong et al., 2024). JA and SA-dependent signal responses to pathogen attack require transcriptional reprogramming, which includes transcriptional factors such as WRKY. In Arabidopsis, WRKY factors act in a complex defense response network as both positive and negative regulators. At present, 79 WRKY genes have been found in Arabidopsis. Notably, AtWRKY33 functions as a positive regulator of resistance toward the necrotrophic fungi *Alternaria brassicicola* and *Botrytis cinerea*, and AtWRKY53 and AtWRKY70 both positively modulated SAR (Wang et al., 2006; Zheng et al., 2006). SA biosynthesis and SA-dependent defenses are regulated by WRKY TFs. BnWRKY expression was examined in response to *S. sclerotiorum* infection, and He et al. found that BnWRKY33 expression was increased 12-fold within one hour following treatment with a rapid alkalinization factor (RALF) associated with *S. sclerotiorum*, further demonstrating the role of WRKY transcription factors in response to attack by necrotrophic pathogens (He et al., 2016; Liu et al., 2018). However, when studying the interactions of BnWRKYs in response to *S. sclerotiorum* infection, Zhang et al. found that while BnWRKY33 expression spikes during early infection, BnaA03.WRKY28 responds by binding to the promoter of *BnWRKY33*, decreasing its expression, resulting in a shift away from defense and back towards growth and development. In this way, they determined that BnaA03.WRKY28 negatively regulates defense to *S. sclerotiorum* (Zhang et al., 2022a). Further studies need to be conducted to identify the precise regulation of TFs in response to *S. sclerotiorum* infection. Identification and characterization of *AtWRKY53* and *AtWRKY70* homologs in *B. napus* could provide insight into improving resistance against necrotrophic pathogens.

### 5.5 Phytohormones

Phytohormone signaling in plants drives growth and development as well as response to biotic and abiotic stressors (Bari and Jones, 2009; Denancé et al., 2013). As small signaling molecules, phytohormones direct the expression of countless genes within plants (Xia et al., 2015). There are many classes of plant hormones which are categorized by their chemical structures and role within the plant. Of the many plant hormones, salicylic acid (SA), jasmonic acid (JA), and ethylene (ET) are commonly associated with defense signaling in plants, where SA is traditionally associated with response to biotrophic pathogens and JA/ET signaling are associated with necrotrophic pathogen response (Jones and Dangl, 2006). However, given the transient biotrophic phase of *S. sclerotiorum*, both pathways are likely crucial for improving resistance. A study conducted in 2014 measured the hormone levels within *B. napus* following infection by *S. sclerotiorum*, and they observed that infected plants produced significantly elevated levels of SA at 12, 24, and 48hpi. Further, they observed a slight decrease in JA production at 12hpi, followed by a significant increase at 24hpi (Nováková et al., 2014). These findings demonstrate that both pathways are induced following infection. To further explore the role of SA and JA in defense response, they pretreated plants with SA or JA derivatives to induce these hormone pathways prior to infection. Interestingly, contrary to the traditional roles of SA and JA, they observed that SA induction significantly improved the resistance response in detached leaf assays, where only a slight and non-significant decrease in infection was observed in the JA pretreatment group. The results from this study exemplify two key points: first, this demonstrates the importance of phytohormones in plant disease response, and second, phytohormones are capable of producing different reactions in different pathosystems and oversimplifications stating that SA and JA are only meaningful in biotrophic and necrotrophic responses, respectively, need to be reevaluated for each host-pathogen interaction being assessed.

## 6 Defense mechanisms of sunflower to *S. sclerotiorum*

In contrast to soybean and canola interactions with *S. sclerotiorum*, this pathogen causes three distinct diseases in sunflowers. *S. sclerotiorum* can infect the flower directly, resulting in head rot (SHR), the stem causing mid-stalk rot (MSR), or it can infect through the roots, resulting in basal stalk rot (BSR) (Gulya et al., 2016; Harveson et al., 2016). While *S. sclerotiorum* is not the greatest threat to crop loss in sunflower production, it is capable of causing up to 50% yield reductions in severely infected fields (Research: USDA ARS, 2022). Similar to soybean and canola, current strategies for combating these three diseases rely primarily on the use of fungicides, rotations, and avoidance. However, the prevalence of the disease in sunflower fields remains a significant problem as genetic resistance to HR, MSR, and BSR are thought to be physiologically distinct, and developing resistance to one disease is not likely to protect against the others in infected fields (Talukder et al., 2014).

Interestingly, several wild *Helianthus* relatives have been identified, each displaying variable tolerance levels to the three sunflower disease complexes. Still, none of the varieties display resistance to all three complexes, and differences in *Helianthus* ploidy levels hinder traditional resistance breeding.

### 6.1 Metabolites and intermediates

Plant metabolites and intermediates from the citric acid cycle (TCA) serve as the precursors for several key amino acids, cell wall molecules, and phytohormones (Scheideler et al., 2002; Rhoads et al., 2006; Zhu et al., 2008). The differential responses of plant metabolite production in response to pathogen attack can offer essential insights into plant response, as well as comparisons between susceptible and resistant responses. Peluffo et al. found key differences in resistant and susceptible sunflower varieties in their metabolite production in response to *S. sclerotiorum* infection. Their analysis found that a resistant line RHA801 produced significantly higher levels of trehalose, inositol, glycerate, citrate, isocitrate, and succinate compared to the susceptible line HA89. Interestingly, the susceptible line showed increased production of tyrosine and chlorogenate, which are associated with the production of phenolic compounds, auxins, and SA. In correspondence with these results, they also found a significant decrease (nearly 50%) in phenylalanine ammonia-lyase activity in RHA801 at 1-day post-inoculation compared to mock-inoculated plants, indicating a shift away from the phenylpropanoid biosynthesis pathway (Peluffo et al., 2010).

### 6.2 Transcription factors and non-coding RNAs

Using RNA-seq analysis, DEGs were identified in susceptible and moderately resistant lines. Some key DEGs identified in the tolerant lines were the transcription factor IBH1, while another was a non-coding RNA (Fass et al., 2020). In *Arabidopsis*, the IBH1 TF was determined to inhibit a different transcription factor, HBI1, which functions in the promotion of growth and inhibition of immune response (Fan et al., 2014). Therefore, it can be understood that the IBH1 TF facilitates the transition between growth and development and immune response, although more work needs to be done to confirm that these molecular mechanisms are equivalent across the different hosts. The ncRNAs were the most consistent DEG signal found in the study by Fass et al., 2020. Although this research did not test the role these ncRNAs play in disease response. However, it is well established that ncRNAs function as genetic regulators in addition to serving as precursors to sRNAs, microRNAs, and small-interfering RNAs, all of which have known defense response roles in other systems.

Further, they identified a hypoxia-responsive protein. This family of proteins is thought to be associated with group VII Ethylene response factors, which can be understood to be related to necrotrophic pathogen response signaling (Fass et al., 2020).

### 6.3 Pathogenesis-related proteins

Pathogenesis-related proteins are a well-known group of proteins that play a crucial role in plant defense and were first discovered in tobacco by Van Loon and Van Kammen in 1970 (van Loon, 1985). To date, 16 recognized families of PR proteins have been identified, grouped by their sequence similarity and enzymatic activities (Edreva and Kostoff, 2005). Proteins within the PR-5 group are believed to contribute to biotic defense by disrupting the pathogens’ plasma membrane permeability and are known to localize in various tissues, including roots and flowers (Sudisha et al., 2012; Musa-Khalifani et al., 2021). In a study comparing PR5 gene expression in susceptible and moderately resistant accessions of sunflowers, it was found that PR5 showed a 5-fold increase in expression in the resistant lines 3 hours after infection, implicating the role of PR5 in defense against *S. sclerotiorum* attack (**Table 2**) (Musa-Khalifani et al., 2021). While the utility of this PR protein is clear, there are still many more PRs of sunflower that require identification and functional analysis in the search for improved defense strategies against white mold. Another important group of PR proteins includes the germin-like proteins (GLPs). Germin-like proteins comprise a large class of water-soluble plant proteins that are known to play important roles in plant defense response to biotic and abiotic stressors (Zhang et al., 1995; Schweizer et al., 1999). To date, 16 HaGLPs have been suggested, based on EST motifs, although they have not all been confirmed and only two have been characterized for function (Beracochea et al., 2015). A sunflower GLP, HaGLP1 was first described in 2003 by Fernandez et al. in the search for resistance-related genes to Sclerotinia head rot (Fernández et al., 2003). The function of HaGLP1 was functionally studied in 2015 where Beracochea et al. transformed *Arabidopsis thaliana* with the HaGLP1 gene and challenged them with *S. sclerotiorum.* While both the transgenic and wild-type plants showed notable lesion expansion, the transgenic lines showed statistically improved disease response compared to the WT plants. It was further determined that the expression of HaGLP1 in *A. thaliana* resulted in increased production of O_2_^-^ and H_2_O_2_, demonstrating its role in the generation of reactive oxygen species in the presence of *S. sclerotiorum* (Beracochea et al., 2015). Another GLP, HaGLP3, was reported in an association mapping study conducted by Filippi et al. in 2020. In this study, the marker for this gene was found to be associated with increased resistance response to Sclerotinia head rot, although functional studies were not conducted (Filippi et al., 2020). Future work should focus on the discovery of other GLPs in sunflower, along with functional studies to elucidate the mechanism by which HaGLPs modulate resistance responses.

### 6.4 Phytohormones of helianthus

Phytohormone signaling in plant defense response has been well characterized in many systems, where it is generally accepted that hormones such as salicylic acid (SA) are utilized against biotrophic pathogens to induce local immune responses and initiate systemic acquired resistance (Bari and Jones, 2009; Denancé et al., 2013). Other hormones such as jasmonic acid (JA) are also used in pathogen defense signaling, commonly suggested in the response to herbivory and necrotrophic pathogens, which result in transcriptional reprogramming and biosynthesis of defense-related secondary metabolites. Another important phytohormone includes abscisic acid (ABA). Abscisic acid is commonly discussed in the context of plant growth regulation, stomatal conductance, and response to abiotic stressors. However, it also plays a crucial role in pathogen defense, as ABA pathways are highly interconnected with SA and JA signaling pathways (Rai et al., 2024). In a study comparing the phytohormone responses of resistant and susceptible lines of sunflower following *S. sclerotiorum* infection, it was determined that following infection, ABA levels continuously increased in resistant lines, where susceptible lines showed a strong peak at 12 hpi, followed by a drop in ABA below that of the untreated controls. When observing SA production, the resistant line showed significant increases from the control at 24hpi, followed by a return to untreated levels. Interestingly, the SA levels in the susceptible line were decreased at nearly every time point and were significantly lower than the controls, demonstrating a positive correlation between SA and ABA (Liu et al., 2017). This data further demonstrates the importance of SA signaling during the early stages of infection. In assessing the role of JA in defense response, Monazzah et al. assessed the expression levels of the helianthus plant defensin gene (*PDF1.2*) in resistant and susceptible varieties following infection. PDF1.2 is considered a marker gene for the JA/ET pathway and can be used as a proxy for indirect measurement of JA production. Their results demonstrated that the partially resistant varieties had significantly increased expression levels of *PDF1.2* at 48 and 72hpi, where the susceptible variety showed significant downregulation at those same time points. It can be understood that the ability of the resistant lines to properly initiate a JA/ET response confers a stronger defense response in this system (Monazzah et al., 2018b).

## 7 Conclusion

Taken together, individual plant families have developed both common and specific strategies to combat SSR, potentially holding a repository of undiscovered quantitative resistance genes. However, complete resistance to *S. sclerotiorum* is unknown in any of its hosts, driving the need for comparative studies of these important interactions. In this review, common defense strategies of these hosts were evaluated, demonstrating overlapping defense strategies such as the use of phenylpropanoid intermediates, PGIPs, and phytohormones. Additionally, host specific defense strategies were explored such as glyceollins and glucosinolates, found in soybean and canola, respectively. In contrast, sunflowers were not found to have any species/family specific defense strategies. In fact, they are unique in their susceptibility compared to the other two hosts, being vulnerable to three diseases on different tissue regions. A study conducted in 2022 aiming to elucidate the root susceptibility of sunflower, found that root infection by *S. sclerotiorum* is specific to plants of the Asteraceae family, while hosts outside of this family such as canola and dry bean were able to halt mycelial spread to the stem following root infection.
